# Why Cows Fail to Breed, and What to Do with Them

**Published:** 1898-04

**Authors:** George B. Jobson

**Affiliations:** President of the Pennsylvania State Veterinary Medical Association, Franklin, Pa.


### WHY COWS FAIL TO BREED, AND WHAT TO DO WITH THEM.

By George B. Jobson, V.S.,

PRESIDENT OF THE PENNSYLVANIA STATE VETERINARY MEDICAL ASSOCIATION, FRANKLIN, PA.

The subject which your worthy President has assigned to me, and which he has put in the form of a question, "Why cows fail

[1] Read at the annual meeting of the Pennsylvania Jersey Cattle-Breeders' Association, March, 1898.

to breed, and what to do with them?" is in many respects a difficult question to answer, and one to which it is impossible to give a direct categorical reply. You will understand why this is so when I say that sterility does not always assume the same form, may result from various causes, and that these causes are frequently obscure and difficult of diagnosis. A disordered condition of the procreative organs, proceeding from disease, malformation of these organs, or it may be causes extraneous to the individual female, may result in barrenness, and as there is no one particular form of this disease (for such it may be termed), so there cannot be any specific remedy. However, it matters not what may be the cause of barrenness, there is no gainsaying the fact that the loss caused by the failure of some particularly choice cows to breed is one of the most annoying to which the breeder of high-bred stock is subjected.

Highly bred, pampered cows, getting little or no exercise, and a liberal allowance of grain, will not prove as sure breeders as the ordinary run of farmers' cattle. Yet it is sometimes a difficult matter to get those in charge of a herd to understand the necessity for giving dairy cattle exercise during winter; they will tell you turning them out makes them look rough; that there will be a falling off in the milk-supply, and give you sundry reasons, satisfactory, at least, to themselves, why they are much better off shut up in the stable than turned out for exercise. Supposing we admit that cows turned out in winter do look a little rougher in the coat than those which are closely shut up in a warm stable, or that they may give a little less milk, this is more than compensated in the better health of the herd, and I will state right here that some of the Jersey cows which have made the largest yearly milk records were regularly exercised by being led one mile daily in winter, oftentimes in the very roughest kind of weather.

Where failure to conceive results from obesity and want of exercise, the remedy is obvious: shut off the supply of grain and turn the cow out regularly every day; while an occasional purgative of sulphate of magnesia will prove beneficial in ridding the system of the superfluous fat. Of course, where there is complete fatty degeneration of the uterine appendages there can be no remedy; but where, as is more usually the case in young cows, there is only fatty infiltration of the cells of the ovaries and walls of the Fallopian tubes, leading to inactivity of the former organs, and blocking or occlusion of the tubes, the excessive deposition of fat can be reduced by letting your cows rough it in winter, and in summer turning them out on bare pasture.

A herd the members of which are closely inbred is likely to have more barren females than one having frequent and strong out-crosses. Take, for example, the Bates Shorthorn herd, the Duchess family of which was notoriously inbred, a large percentage of which proved non-breeders or failed to produce living offspring. Should you wish to intensify certain characteristics by resorting to inbreeding, and where certain cows fail to conceive when mated with a bull raised in the same herd and closely related to them in blood, if you do not wish to use an outcross, get an animal from a distance, bred in collateral lines, the difference in soil and climate in two or three generations so changing the constitution that you will get precisely the same results as from an outcross. The cause in this case is really extraneous and not inherent in the cow, she proving fertile by a change in mating. We might take another case where the cow is not at fault. Probably you have an old bull which has made a splendid record as a producer of great butter-cows, and you may have reserved him to put to a few of your choicest females, yet to your disappointment they fail to breed, showing that his procreative powers are on the wane through age and, probably, over-service. The trouble in this case proceeds from seminal weakness or want of vitality in the spermatozoa of the male, and its failure to properly impregnate or vitalize the ovulum of the female. This defect can sometimes be overcome by using tonics adapted to the generative organs of the male, such as the preparations of phosphorus, iron, etc. A very good prescription given me by a medical friend, and which has been used with good effect on bulls and stallions, is :

℞.—Pyrophosphate of iron . . . . . ℥jss
Phosphide of zinc . . . . . . grs. xlviij
Nux vomica . . . . . . . ℥j

Mix and divide into twenty-four powders. Give one powder three times a day in feed.

I have alluded to diseases of the procreative organs causing sterility. We will consider a few of the more common forms of these uterine diseases. A retained placenta or after-birth, when not removed within a day or two after calving, will prevent conception, either from the fetid, sanious discharge consequent on the placental membranes being allowed to rot away piecemeal, causing irritative inflammation of the uterus and vagina, or it may drift on to leucorrhœa. In either case, or where leucorrhœa is the result of injuries received during parturition, a good remedy for this morbid condition of the organs is creolin (one part to one hundred parts of water), which makes a good antiseptic wash, and not so irritating as carbolic acid. Irrigate the womb with this thoroughly once a day. Use a syringe with a piece of rubber tubing, bare your arm, wet it with the creolin solution (which ought to be about blood-heat), put your hand into the vagina, making sure the tubing of the syringe enters the womb, and use a liberal amount of the wash. As the discharges in leucorrhœa are acid, and the spermatozoa will not live in an acid medium, it is very good practice to use a solution of borax (one-half ounce to one quart of warm water) following the creolin wash. A course of iron and bitter tonics ought also to be given internally.

Occlusion or closure of the mouth and neck of the uterus renders conception mechanically impossible, the semen of the male failing to enter and fertilize the ovum. This may be spasmodic, but more frequently is the sequel to an injury to these parts received during parturition. Various devices in the shape of dilators have been invented to overcome this difficulty. One of the simplest and safest methods is to bare the arm, pare the nails to prevent wounding the parts, smear the hand and arm with a little lard or vaseline, and use the index-finger as a dilator. The insertion of the finger full length is all that is necessary. Should it be so firmly closed that it is impossible to enter the finger, smear the mouth of the womb with a little belladonna ointment and try again after a few hours. In performing this operation, always go slowly and take plenty of time, the fibres of the mouth being very resistant and almost cartilaginous, and should you tear the parts adhesive inflammation takes place in healing, leaving the parts in worse condition than at first. I have used with good effect tents made out of sea-tangle, or what is preferable, made out of soft, porous wood thoroughly dried. After insertion the moisture from the parts causes the tent to swell and act as a dilator. These tents were inserted into the neck of the womb two or three days before we expected the cow to come in heat, were allowed to remain in all night, and after being removed a warm alkaline wash of borax or soda was used to allay any irritation caused by the introduction of the tent until the cow came in heat.

Some stock-owners follow the very reprehensible practice of breeding large and vigorous bulls to undersized heifers. This is frequently productive of injury to the mouth of the womb, followed by induration. Instances have occurred where heifers have been injured in the back and limbs, and the point of the hip knocked down by being thrown violently to the ground.

Ovarian dropsy or tubercular deposits in both ovaries will prevent conception, while a cow with tubercular disease of the uterus, the ovaries not being infected, may conceive, but will surely abort. Where a bull is used on a cow in this condition there is great danger of him conveying the disease to other cows. In fact, a cow which frequently aborts ought always to be viewed with suspicion, and, even if there are no visible signs of disease, the safer way is to destroy her.

Occasionally cows will come in heat, take the bull, miss one or two periods, and then côme in heat again. These I believe are in some cases really abortions. I recollect picking up behind a cow six weeks in calf a fœtus about the size of a very small newly-hatched sparrow. Had this happened out of doors, or had it been dropped in the manure, it would have passed unnoticed, as there was no discharge from the vagina or other visible signs of disturbance. In cases such as these fluid extract of blackhaw (*vibirnum prunifolium*) will prove useful in doses of from a half to one ounce daily for three or four weeks, it being a most excellent uterine tonic.

A cow which has aborted ought immediately to be separated from the herd, irrigated thoroughly with the creolin solution once a day for at least one week, and until all vaginal discharge has ceased, before being again served by the bull or allowed to mix with the herd.

I have referred to malformation of the procreative organs being a cause of sterility. This rarely occurs except where a heifer is twin sister to a bull. In these instances the female is barren, the internal organs being usually hermaphrodite and not properly formed. In the beef breeds they somewhat resemble steers about the head and neck ; in the Jersey breed less so, and except it may be a very small and contracted vagina, there may be no outward indications of anything abnormal.

In reviewing the whole question it may be stated as an axiom that the more artificially cattle are kept, and the more we diverge from nature's methods, the larger will be the percentage of barren animals. We find it so in all the varieties of the domesticated animals. My lady's lazy, fat canine pet, as well as prize swine, sheep, and cattle, overfed and kept for purposes of exhibition, will not prove as sure breeders as those kept under more natural conditions. If we transgress nature's laws to any extent, in this or any other respect, we must pay the penalty. Neither, on the other hand, is the starvation system, pursued during winter by too many farmers, advocated, whereby dairy cows are so much reduced in condition that it takes a large part of summer for them to recuperate and recover lost ground, but that happy medium and system of management which, combined with a rational system of breeding, will give each individual member of the herd a vigorous constitution. Follow the rules of hygiene in your stables, give plenty of ventilation, plenty of light, admitting all the sunlight possible; keep the cattle and stalls clean, white-washing the latter frequently; give an abundance of pure water and a moderate allowance of food, turning the cattle out daily for exercise; and lastly, by all means do not heat your stables with steam; many stock-owners have done so to their sorrow, and paid the penalty in the weakened vitality of their stock. Above all things, a good dairy cow must have a strong constitution to stand the double drain on her sytem of producing offspring and giving a large yield of milk; therefore, do not adopt a systematic course of continued in-breeding, do not breed from immature animals, nor from animals in their decadence, and do not breed from animals below par, which are weak in constitution and obviously lacking in vital stamina, however rich in blood, or however well bred they may be. Get rid of these at the earliest opportunity, and let the other fellow have the blood.

If you adopt the methods which I have recommended relative to the hygiene of the herd and keeping the cow-stable in sanitary condition, weeding out all inferior animals, irrespective of their breeding, your herd will be comparatively free from disease; you will have little trouble with barren cows or abortions, and your herd will be a source of satisfaction to yourself and a credit to your system of management.

